# Kindlin-2 Mediates Mechanical Activation of Cardiac Myofibroblasts

**DOI:** 10.3390/cells9122702

**Published:** 2020-12-17

**Authors:** Elena Godbout, Dong Ok Son, Stephanie Hume, Stellar Boo, Vincent Sarrazy, Sophie Clément, Andras Kapus, Bernhard Wehrle-Haller, Leena Bruckner-Tuderman, Cristina Has, Boris Hinz

**Affiliations:** 1 Laboratory of Tissue Repair and Regeneration, Faculty of Dentistry, University of Toronto, Toronto, ON M5G 1G6, Canada; elgodbout@ohri.ca (E.G.); d.son@utoronto.ca (D.O.S.); stephanie.hume@utoronto.ca (S.H.); hboo@alumni.uwo.ca (S.B.); vincent.sarrazy@unice.fr (V.S.); 2 Division of Clinical Pathology, University Hospital, University of Geneva School of Medicine, 1211 Geneva 4, Switzerland; Sophie.Clement@unige.ch; 3 Keenan Centre for Biomedical Science, St. Michael’s Hospital, Toronto, ON M5B 1W8, Canada; andras.kapus@unityhealth.to; 4 Department of Surgery, University of Toronto, Toronto, ON M5T 1P5, Canada; 5 Department of Cell Physiology and Metabolism, Faculty of Medicine, Centre Médical Universitaire, University of Geneva, 1211 Geneva 4, Switzerland; Bernhard.Wehrle-Haller@unige.ch; 6 Medical Center and Medical Faculty, University of Freiburg, 79104 Freiburg, Germany; bruckner-tuderman@uniklinik-freiburg.de (L.B.-T.); cristina.has@uniklinik-freiburg.de (C.H.)

**Keywords:** fibrosis, focal adhesion, mechanical stress, nuclear shuttling, mechanosensation

## Abstract

We identify the focal adhesion protein kindlin-2 as player in a novel mechanotransduction pathway that controls profibrotic cardiac fibroblast to myofibroblast activation. Kindlin-2 is co-upregulated with the myofibroblast marker α-smooth muscle actin (α-SMA) in fibrotic rat hearts and in human cardiac fibroblasts exposed to fibrosis-stiff culture substrates and pro-fibrotic TGF-β1. Stressing fibroblasts using ferromagnetic microbeads, stretchable silicone membranes, and cell contraction agonists all result in kindlin-2 translocation to the nucleus. Overexpression of full-length kindlin-2 but not of kindlin-2 missing a putative nuclear localization sequence (∆NLS kindlin-2) results in increased α-SMA promoter activity. Downregulating kindlin-2 with siRNA leads to decreased myofibroblast contraction and reduced α-SMA expression, which is dependent on CC(A/T)-rich GG(CArG) box elements in the α-SMA promoter. Lost myofibroblast features under kindlin-2 knockdown are rescued with wild-type but not ∆NLS kindlin-2, indicating that myofibroblast control by kindlin-2 requires its nuclear translocation. Because kindlin-2 can act as a mechanotransducer regulating the transcription of α-SMA, it is a potential target to interfere with myofibroblast activation in tissue fibrosis.

## 1. Introduction

The ability of cardiac fibroblasts to sense and control the mechanical properties of the extracellular matrix (ECM) is essential to adapt heart tissue to mechanical load (e.g., hypertension) and to repair injuries (e.g., after myocardial infarct) [1,2]. Aberrant mechanosensing results in activation of cardiac fibroblasts into α-smooth muscle actin (α-SMA)-expressing myofibroblasts that are characterized by excessive collagen secretion and contraction [3,4,5,6]. The outcome of myofibroblast dysfunction is fibrosis—accumulation of stiff collagen scar tissue. Stiff fibrotic tissue impairs heart distensibility, pumping and valve function, contributes to diastolic and systolic dysfunction, and affects myocardial electrical transmission, leading to arrhythmia and ultimately heart failure [7,8,9,10].

Fibroblasts transmit and perceive forces via transmembrane integrins in focal adhesions (FAs) [11,12,13,14]. Within FAs, kindlins directly bind the cytoplasmic tails of β1 and β3 integrins and, together with talin, co-activate integrins to regulate cell–ECM adhesion and actin dynamics [15,16,17,18,19,20,21,22,23,24,25]. The kindlin protein family consists of three evolutionary conserved members, kindlin-1, -2, and -3 [26]. Kindlin-1 is exclusively expressed in epithelial cells and kindlin-3 is restricted to hematopoietic cells [18,27,28], while fibroblasts express only kindlin-2, also known under the gene name fermitin family homolog 2 (*FERMT2*). In contrast to kindlin-1 and kindlin-3, which are well studied in the context of disease [29,30], our current understanding of the pathophysiological functions of kindlin-2 is limited.

Kindlin-2 is expressed in the heart, lungs, and skin [31,32] and is known to control integrin affinity, mediate binding to contractile stress fibers, and regulate integrin trafficking [15,16,18,20,21,22,33,34]. Kindlin-2 regulates the ability of human dermal fibroblasts to migrate and to contract collagen gels which are essential mechano-biological functions of fibroblasts in conditions of tissue repair and fibrosis [35]. More recently, kindlin-2 was also shown to play a role in fibrotic processes in hepatic stellate cell activation [36]. However, the mechanisms of mediating these actions are incompletely understood. We here provide evidence that kindlin-2 can act as a nuclear shuttling protein that regulates the mechanical activation of primary human cardiac fibroblasts (hCF) into myofibroblasts by controlling expression of α-SMA.

## 2. Materials and Methods

### 2.1. Animal Model of Induced Heart Fibrosis

Fibrosis was produced by inducing cardiac hypertrophy in rat hearts as previously described [37]. Briefly, silk ligature was placed on the free dissected abdominal aorta between the two renal arteries, using a coarctation diameter of 0.4 mm that induces robust interstitial fibrosis in the left ventricles over 4 weeks. Hearts were dissected and cryo-embedded to produce sections of 5 µm thickness. Authorizations were obtained from the Ethical Committee of Geneva Medical faculty (Protocol #31.1.1005/1115/II) and from the Animal Care Committee of the University of Toronto (Protocol #20009319).

### 2.2. Cell Culture

Commercially available primary hCF isolated from right ventricles of human male donors (age 39–42 years) (CC-2904, Lonza, Walkersville, MD, USA) was expanded in complete FGM-3 medium (Lonza) to passage P3 and routinely cultured between passages P3-P5 in medium consisting of 45% Dulbecco’s modified Eagle’s medium (DMEM), 45% F12 (Gibco Invitrogen, Burlington, ON, Canada), 10% fetal calf serum (Invitrogen), 1000 U/mL penicillin/streptomycin (Invitrogen), and fibroblast growth supplement (ScienCell, Carlsbad, CA, USA). Rat embryonic fibroblasts (REF) and human MRC-5 cells were used for selected experiments and cultured in Dulbecco’s modified Eagle’s medium (DMEM), 10% fetal calf serum (Gibco), 1000 U/mL penicillin/streptomycin (Invitrogen). Soft silicone culture substrates (ExCellness Biotech, Lausanne, Switzerland) were used to mimic the elastic range of healthy and diseased tissue and to control myofibroblast activation. We selected 3 kPa soft, 26 kPa medium-stiff, and 65 kPa stiff substrates to model increasing severity of fibrotic scar stiffening [38]. Silicone culture substrates were oxygen plasma-treated and coated with 2 μg/cm^2^ fibronectin (Millipore, Billerica, MA, USA) to promote cell attachment [39].

### 2.3. Quantification of Cell Contraction

Cells were seeded at a concentration of 2500 cells/cm^2^ for 8 h onto fibronectin-coated (2 μg/cm^2^) silicone substrates with surfaces activated to observe wrinkle formation upon cell contraction, as described previously [40]. Phase contrast and epifluorescence images were acquired with an inverted microscope (Axiovert 135, Carl Zeiss). To induce cell contraction, cells were treated with 20 μM plasminogen activator inhibitor (PAR)-1 agonist TFLLRN (Anaspec, Fremont, CA, USA) for 1 h. To quantify the percentage of contractile cells, the numbers of wrinkling and total cells were counted for ≥10 random fields of view (~25 cells/field) per experimental condition in three experimental repeats.

### 2.4. Mechanical Cell Stimulation

To subject hCF to global strain, we used custom-made stretchable culture membranes that were functionalized with 2 μg/cm^2^ fibronectin (Millipore) [39]. HCF were cultured at 1000 cells per cm^2^ for 24 h before straining the cultures by 20% in a custom-built uniaxial stretching device for 1 h. To strain hCF at sites of FAs, we used magnetic microparticles (3 µm diameter, Fe_3_O_4_, Sigma, Oakville, ON, Canada), coated with 100 μg/mL fibronectin (Millipore) [35,41]. Fibronectin-coated microbeads were added to hCF cultures for 30 min, followed by three washes with medium to remove unbound microbeads. A ceramic permanent magnet was then applied to generate tensile forces perpendicular to the cells’ growth plane via the attached microbeads for 1 h. Microbead-bound proteins were isolated by washing cells with PBS and scraping into extraction buffer (0.5% Triton X-100, 50 mM NaCl, 300 mM sucrose, 3 mM MgCl_2_, 10 mM 1,4-Piperazinediethanesulfonic acid, pH 6.8), supplemented with protease inhibitors cocktail (Sigma). The suspension was sonicated and the microbead fraction was separated from the cytosolic fraction using the magnetic separation stand. Bead fractions were suspended in ice-cold extraction buffer, homogenized, and boiled in sample buffer for 5 min. Microbeads were pelleted and supernatant was collected for Western blotting.

### 2.5. Immunostaining, Antibodies, and Microscopy

Cells were fixed with 3% paraformaldehyde and permeabilized with 0.2% Triton X-100. Tissue sections were fixed with acetone at −20 °C. Samples were stained with primary antibodies directed against kindlin-2 (rabbit [42], and mouse clone 3A3, Millipore), lamin B (ab16048, Abcam, Toronto, ON, Canada), vimentin (M0725, Dako, Burlington, ON, Canada), α-SMA (αSM-1, a kind gift of Dr. G. Gabbiani, University of Geneva, Geneva, Switzerland), vinculin (V9264, Sigma), talin (T3287, Sigma), β-cytoplasmic actin (13E5, Cell Signaling Technology, Danvers, MA, USA), Smad2/3 (8685, Cell Signaling Technology), phospho-Smad2/3 (882S, Cell Signaling Technology), and fibronectin (F3648, Sigma). TRITC-conjugated goat anti-mouse IgG1 (Jackson Laboratories, Bar Harbor, MA, USA), Alexa647-conjugated IgG2a, and Alexa488-conjugated goat anti-rabbit (Invitrogen) were used as secondary antibodies, and 4′,6-diamidino-2-phenylindole (DAPI) as nuclear stain. Micrographic images were taken using an upright epifluorescence microscope (Zeiss Axio Observer M35, Carl Zeiss, Jena, Germany) equipped with an Axiocam HR camera (Carl Zeiss). For quantification of kindlin-2 nuclear and cytosolic staining, epifluorescence microscopy images were taken using optical sectioning by structure illumination (Apotome, Carl Zeiss) and analyzed using the region of interest overlay function of Fiji [43] either on the nucleus (nuclear signal) or as a “doughnut” with an identical area just around the nucleus (cytosolic). For quantification, at least 10 cells per image and at least 5 images per experimental condition were analyzed from at least three independent experiments. Scanning confocal images were acquired using a Leica TCS SP2 AOBS confocal microscope. All images were assembled using Adobe Photoshop. Contrast and brightness were enhanced identically over all images for publication purposes.

### 2.6. Nuclear Extracts and Western Blotting

For Western blotting, we produced total cell extracts according to standard procedures, and nuclear and non-nuclear (cytosol and cytoskeleton) extracts using a nuclear extraction kit (Pierce, Thermo Fisher Scientific, Waltham, QC, Canada) according to the manufacturer’s recommendation. Blots were probed with the same primary antibodies as described for microscopy. Horseradish peroxidase-conjugated secondary goat anti-mouse and goat anti-rabbit antibodies (Invitrogen, Burlington, ON, Canada) were used, followed by chemiluminescence (Invitrogen). Loading controls were vimentin for total cell lysates, lamin B for nuclear fractions, glyceraldehyde 3-phosphate dehydrogenase (GAPDH) for cytosol, and fibronectin for microbead fractions. Densitometry analysis was performed with Fiji software [43].

### 2.7. Plasmids, siRNAs, Transient Transfections, Real-Time PCR, and Luciferase Reporter Assays

Cell transfections were carried out with 1 μg plasmid DNA/2 × 10^5^ cells and 100 nM siRNA/2 × 10^5^ cells using electroporation (Neon, Invitrogen) according to the manufacturer’s recommendations. Full length HA-tagged SRF in pCGN plasmid vector was obtained through Addgene (Cambridge, MA, USA) [44]. For overexpression studies, human kindlin-2 cDNA *(FERMT2)* and GFP cDNA were cloned into the Hind III-Xho I sites of pcDNA3 to produce kindlin-2 with N-terminal GFP. Deletion of the kindlin-2 putative NLS (TKKKKKK, amino acids 148–154) from GFP-kindlin-2 was introduced by PCR with the following overlapping primers: 5′-AAAGAAACCCAGAGATCCAAAGCTAGATGACCAGTCTG-3’ and 5′-CAGACTGGTCATCTAGCTTTGGATCTCTGGGTTTCTTT using Pfu Ultra (Agilent), followed by digestion with Dpn I (New England Biolabs) and confirmation by DNA sequencing. Human kindlin-2 specific siRNAs targeting the open reading frame were previously described and characterized: 5′-GCCCUGCAGUAUCAUAUCA-3′ (siRNA K2 I) and 5′-GCCUCAAGCUCUUCUUGAU-3′ (siRNA K2 II) [42]. To knockdown kindlin-2 in REF, 3′-UTR targeting rat kindlin-2 specific siRNA was used: 5′-GCUUGAAGUUAUCGUUUUA-3′ (siRNA K2 III). To trace successfully transfected cells, we used Red-siGlo transfection indicator (D-001630-02, Dharmacon, Burlington, ON, Canada).

Luciferase reporter assays were performed by additionally transfecting cells with a mixture of α-SMA promoter luciferase reporter construct and constitutive renilla luciferase expression vector (Vector pRL-TK, Promega, Madison, WI, USA) as internal control. We used constructs encoding the wild-type and various mutant versions of the 765 bp rat α-SMA (*ACTA2*) promoter controlling the expression of firefly luciferase in pGL3-basic vector [45] Constructs harbored mutations inactivating: (1) the two CArG boxes (CArG-A and CArG-B); (2) the two Smad-binding elements SBE1, SBE2, and the TCE. After 48 h, cells were serum starved for another 3 h and lysed. Luciferase activity in lysates was determined using a dual luciferase reporter assay system kit (Promega) and a multi-well plate luminometer (Berthold Technologies, Harpenden, UK) according to the manufacturer’s instructions. Firefly luciferase activity reporting α-SMA promoter activity was normalized to control renilla luciferase activity of the same sample.

For quantitative real time (qRT) PCR, RNA was isolated using the RNeasy mini kit (Qiagen, SA Biosciences, ON, Canada). qRT-PCR was performed using Superscript Vilo (Invitrogen, Live Technologies, NY, USA) using RT SYBR Green PCR master mix (Qiagen, SA Biosciences, ON, Canada) and forward and reverse primers TGGACGGGATAAGGATGCCA and TGACATCGAGTTTTTCCACCAAC for kindlin-2 *(FERMT2)*; CCCAGACACCAGGGAGTAATGG and TCTATCGGATACTTCAGGGTCA for α-SMA (*ACTA2)*; AGGTCGGTGTGAACGGATTTG and TGTAGACCATGTAGTTGAGGTCA for *GAPDH* on a Step One Plus Real Time PCR System (Applied Biosciences, Live Technologies, Burlington, ON, Canada) according to the manufacturer’s recommendations.

### 2.8. Statistical Analysis

All experiments were performed at least three times and data are presented as mean ±standard deviation (SD) or standard error of the mean (SEM), where applicable. As independent experiments, we considered data obtained from different batches of fibroblasts and/or different animals. We assessed differences between two groups with a one sample Student *t*-test and multiple groups using ANOVA followed by a post-hoc Tukey’s multiple comparison test. Differences were statistically significant with *p* ≤ 0.05.

## 3. Results

### 3.1. Kindlin-2 Expression Is Upregulated in Activated Cardiac Fibroblasts In Vitro and In Vivo

**Figure 1:**
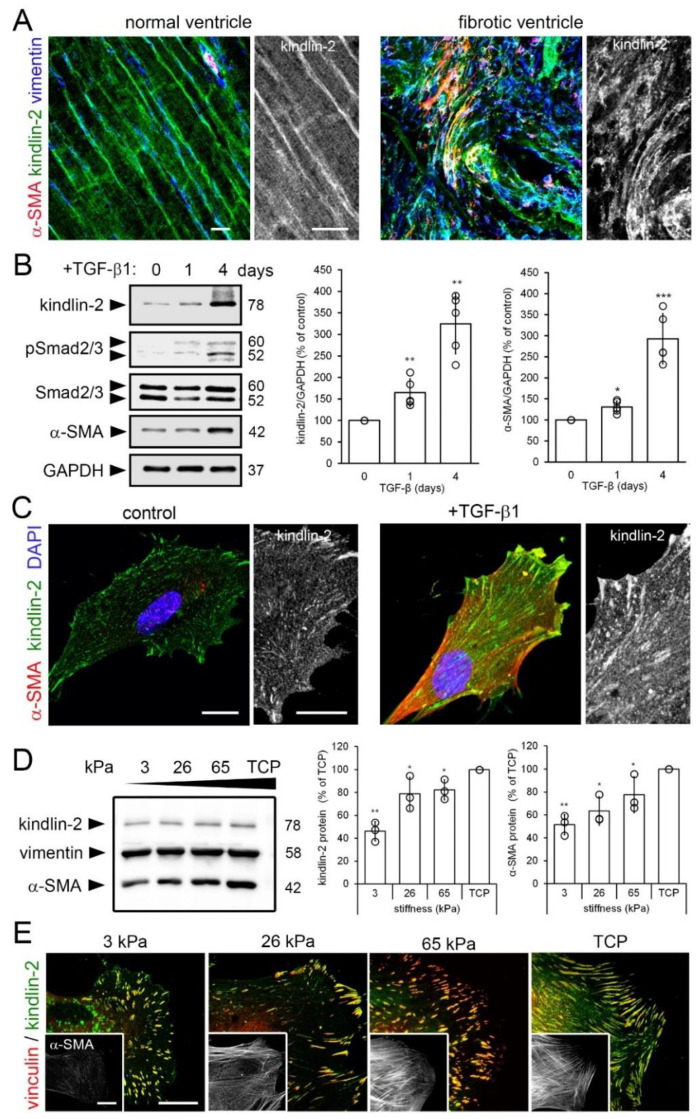
Kindlin-2 expression is enhanced in stiff mechanical environments in vivo and in vitro. (**A**) Sections of normal and hypertrophic rat hearts were stained for kindlin-2 (green), α-smooth muscle actin (α-SMA) (red), and vimentin (blue), and were observed with confocal microscopy; average intensity projections of z-stacks are displayed. Vimentin- and α-SMA-positive myofibroblasts in the hypertensive heart strongly express kindlin-2 in fibrotic lesions. (**B**) Human fibroblasts were cultured on tissue culture plastic (TCP) for 5 day and treated for 1–4 day with transforming growth factor (TGF)-β1 (2 ng/mL) to assess expression of kindlin-2 and α-SMA by quantitative immunoblotting with indicated molecular weights. Glyceraldehyde 3-phosphate dehydrogenase (GAPDH) was used as loading control and Smad2/3 to assess TGF-β1 downstream signaling. (**C**) After 4 day treatment with TGF-β1, cells were immunostained for kindlin-2 (green), α-SMA (red), and nuclei (4′,6-diamidino-2-phenylindole, DAPI, blue) and confocal images were taken. (**D**,**E**) Likewise, human cardiac fibroblasts (hCF) were cultured on silicone culture substrates with elastic modulus of 3, 26, and 65 kPa and GPa-stiff TCP for 5 d, followed by analysis using (**B**) immunoblotting, densitometry and (**C**) immunofluorescence for kindlin-2 (green) and vinculin (red). All immunoblot bands were quantified by densitometry, first normalized to GAPDH loading control and then to TCP control. Shown are mean values from at least three independent experiments (data points) ±SD (* *p* < 0.05, ** *p* <0.01, *** *p* < 0.005, using ANOVA followed by a post-hoc Tukey’s multiple comparison test). All scale bars: 20 µm.

Because kindlin-2 controls the affinity of fibroblast integrins and mediates intracellular binding of the integrin cytoplasmic tail to contractile stress fibers, we investigated its role in hCF mechanosensing and myofibroblast activation in conditions of cardiac fibrosis. Expression of kindlin-2 in the mouse heart and contribution to heart development were described previously [31] but the physiological functions are yet unclear. We localized expression of kindlin-2 within the left ventricles of rats that underwent infra-renal abdominal aortic coarctation to develop hypertension and left ventricular heart fibrosis [37]. In normal left ventricles of control animals, kindlin-2 was expressed in vimentin-positive fibroblasts and α-SMA-positive vascular smooth muscle cells of the interstitial myocardium, in addition to cardiomyocytes (Figure 1A) as previously reported [32,46]. In contrast, the disorganized interstitium of fibrotic left ventricles was characterized by accumulation of α-SMA- and vimentin-positive myofibroblasts expressing high levels of kindlin-2 (Figure 1A). These findings suggest a role for kindlin-2 in normal and excessive tissue repair leading to heart fibrosis.

Two main factors control myofibroblast activation: transforming growth factor beta (TGF-β1) and mechanical stress [47,48]. Fibroblasts cultured on conventional tissue culture plastic (TCP) for a total of 4 day exhibited significantly increased expression of kindlin-2 protein after 1 day (1.7-fold) and 4 day (3.2-fold) of TGF-β1 treatment compared to untreated controls (Figure 1B). Concomitantly, the levels of phosphorylated Smad2/3, indicators of active TGF-β1 signaling, were elevated 1 day and 4 day post TGF-β1 treatment. Increase in kindlin-2 expression after 1 day TGF-β1 treatment was relatively higher than that of α-SMA, which was enhanced after 1 day by 1.3-fold and after 4 day by 3.0-fold following TGF-β1 treatment (Figure 1B). These Western blot results were confirmed by immunofluorescence staining, with higher amounts of kindlin-2 and larger kindlin-2-positive FAs observed after 4 day of TGF-β1 treatment compared to control (Figure 1C).

Next, we used silicone cell culture substrates with controlled stiffness to test whether the mechanical ECM conditions characteristic of heart fibrosis can contribute to increased kindlin-2 expression in myofibroblasts. The elastic modulus of heart muscle was previously determined to range between 10–15 kPa [38,49]. We and others have shown that growth on soft culture substrates (3–5 kPa) suppresses heart myofibroblast activation whereas 26–65 kPa stiff substrates approximating the stiffness of fibrotic heart activate cardiac myofibroblasts [49,50,51,52,53,54]. Expression of kindlin-2 and α-SMA in hCF cultured for 5 day on stiffness-tuned substrates increased 2.1- and 1.9-fold, respectively, from 3 kPa to 65 kPa and were highest in hCF on GPa-stiff TCP (Figure 1D). Kindlin-2 localized to small FAs formed on soft and large FAs formed on stiff substrates at the stress fiber termini of cultured hCF (Figure 1E). Collectively, these results show that kindlin-2 expression is co-regulated with myofibroblast activation. Because regulation of kindlin-2 by TGF-β1 has been established before in various cell types [36,55,56,57,58,59], we continued to investigate the relationship between mechanical stress, kindlin-2, and myofibroblast activation.

### 3.2. Mechanical Stimulation of hCF Results in Kindlin-2 Accumulation in the Nucleus

**Figure 2:**
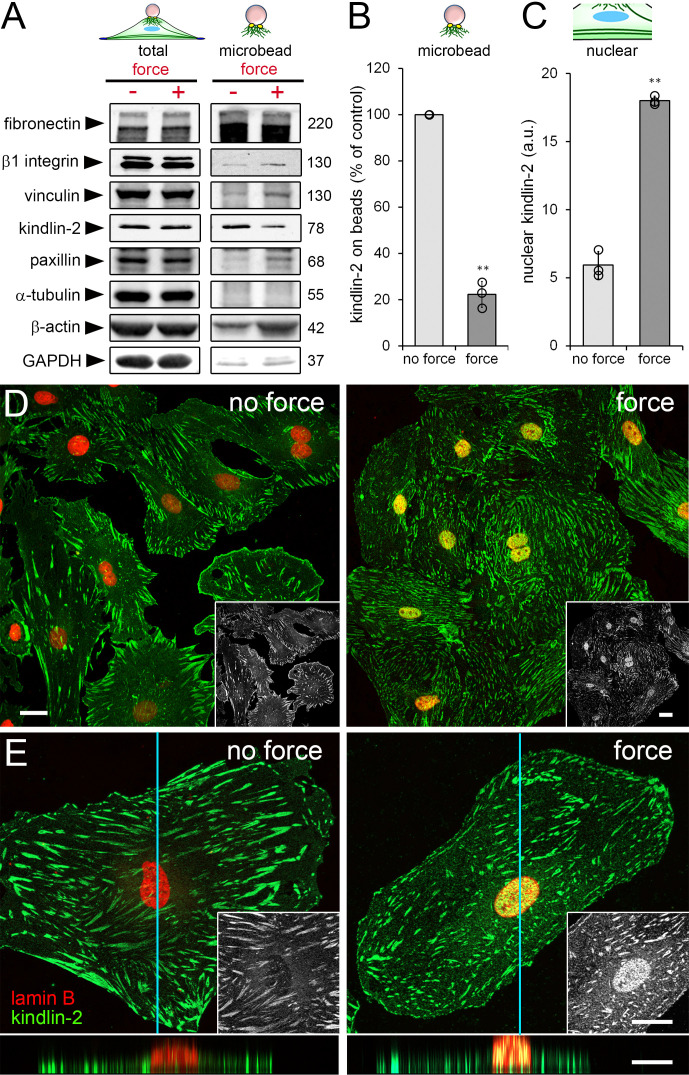
Local application of tensile forces modulates the subcellular localization of kindlin-2. (**A**–**E**) HCF were incubated with fibronectin-coated magnetic microbeads followed by tensile force application using a magnet. (**A**) Total and microbead-associated cell fractions were collected and analyzed by immunoblotting with indicated molecular weights (glyceraldehyde 3-phosphate dehydrogenase = GAPDH) and (**B**,**C**) quantified from band intensities. Fibronectin was used as loading control for microbead-associated fractions. (**D**,**E**) HCF were fixed, permeabilized, stained for kindlin-2 (green) and lamin B (red) and levels of nuclear kindlin-2 (fluorescence intensity, appearing yellow in overlays) were quantified by image analysis. Z-stacks of confocal optical sections were projected into one plane at (**D**) low and (**E**) high magnification. Insets show kindlin-2 staining only, bottom images show optical z-section reconstruction along the indicted plane (blue line) (see also Supplementary Videos S1 and S2). Shown are mean values from at least three independent experiments (data points) ±SD (** *p* < 0.01, using one sample Student *t*-test). All scale bars: 20 µm.

The recruitment of specific cytosolic proteins to FAs under stress is an integral part of fibroblast mechanosensing [12,60,61,62,63]. To assess whether kindlin-2 follows the same regimen, we applied tensile forces to fibronectin-coated ferromagnetic microbeads [35], bound to dorsal FAs of hCF cultured on TCP. Immunoblots of the microbead-associated protein fraction confirmed that the stress-responsive proteins β-cytoplasmic actin, vinculin, paxillin, and β1 integrin were recruited to FAs under strain (Figure 2A, “microbead”).

Unexpectedly, application of strain reduced kindlin-2 levels in the microbead-associated fraction by 4.5-fold compared with non-strained control (Figure 2A); total protein levels did not change (Figure 2A, “total”). Tubulin, fibronectin, and GAPDH were used as controls for loading and fractionation purity. Interestingly, the decrease of kindlin-2 in the strained microbead fraction (Figure 2B) coincided with 3.5-fold enrichment of kindlin-2 in the nucleus of hCF as quantified from confocal immunofluorescence images (Figure 2C). Nuclear localization of kindlin-2 was confirmed in three-dimensional reconstructions of confocal optical sections (Figure 2D,E, Supplementary Videos S1 and S2).

**Figure 3:**
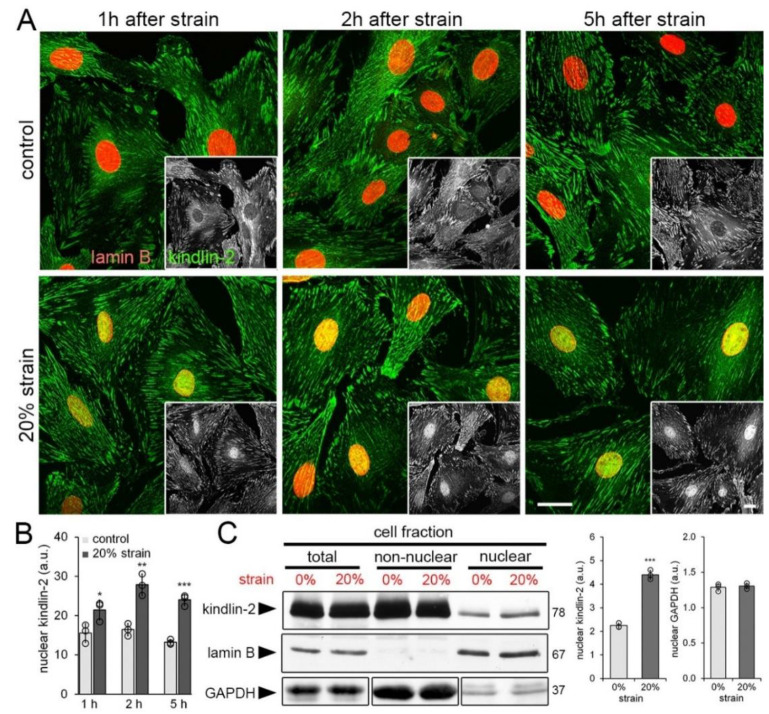
Cell straining leads to kindlin-2 accumulation in the nucleus. Human cardiac fibroblasts (hCF) were strained on silicone culture membranes once by 20% and assessed after different times. (**A**) 1 h, 2 h, and 5 h after unique cell strain (20% strain) or non-strained (control), hCF were immunostained for kindlin-2 (green) and lamin B (red) and scanning confocal images were taken; greyscale insets show kindlin-2. Scale bars: 15 µm. (**B**) Levels of kindlin-2 in the nucleus (fluorescence intensity) were quantified by image analysis from confocal images. (**C**) Nuclear, non-nuclear, and total cell fractions were isolated and analyzed by immunoblotting and densitometry. Lamin B expression levels were used as a loading control for nuclear and total fractions; Glyceraldehyde 3-phosphate dehydrogenase (GAPDH) was used to control similar loading of the non-nuclear and total fractions. Total fractions were diluted 1:2 to allow simultaneous blotting of all fractions without signal saturation. Shown are mean values from at least three independent experiments (data points) ±SD (* *p* < 0.05, ** *p* < 0.01, *** *p* < 0.005, using ANOVA followed by a post-hoc Tukey’s multiple comparison test).

Because the presence of beads was incompatible with centrifuge-based protocols for biochemical protein quantification of nuclear extracts, hCF were next grown on stretchable culture membranes for 4 day and then subjected to one single static strain of 20% to be assessed after different times. Immunostaining (Figure 3A) and quantification of kindlin-2 staining intensity (Figure 3B) revealed increased accumulation of kindlin-2 in the nucleus already 1 h after strain compared with non-strained controls. Kindlin-2 in the nucleus further increased 2 h after strain and decreased moderately after 5 h (Figure 3A,B).

Immunoblotting of the nuclear, non-nuclear, and total cell fractions collected 1 h after 20% cell strain confirmed 2.0-fold increase of kindlin-2 in the nuclear fraction (Figure 3C). Purity of the respective fractions was controlled by immunoblotting for GAPDH that was enriched in the non-nuclear fraction and present at low levels in the nuclear fraction as reported previously [56]. Lamin B was restricted to the nuclear fraction with negligible cross-contaminations (Figure 3C). GAPDH content in the nuclear fraction did not change with strain. Notably, constitutive levels of kindlin-2 in the nucleus were low but present in non-strained hCF grown on stretchable membranes (modulus of 3 MPa) for days (Figure 3A–C), confirming similar observations made with fibroblasts grown on TCP (GPa) and stiff silicone substrates (65 kPa) (Figure 1).

**Figure 4:**
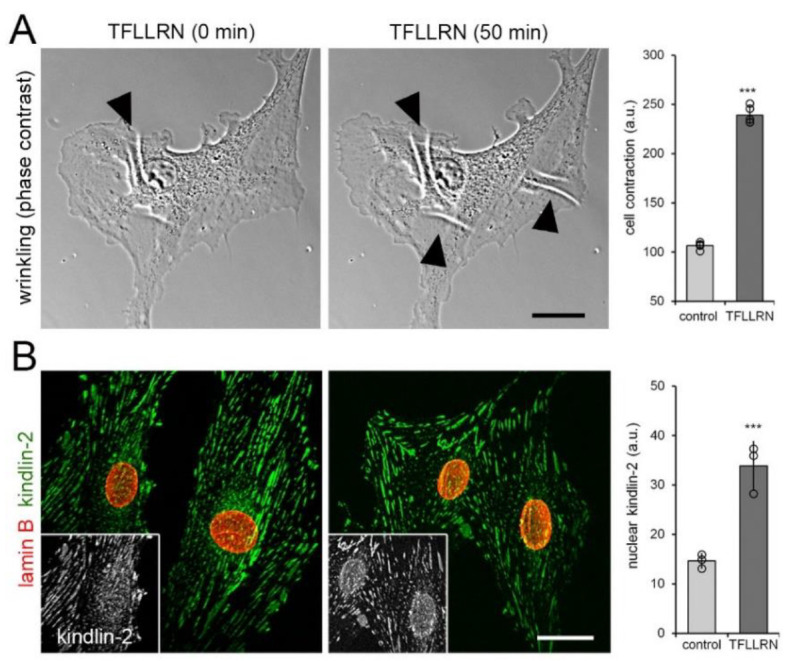
Intracellular force generation leads to kindlin-2 localization to the nucleus. (**A**) Cells grown on “wrinkling” deformable substrates for 8 h were treated with the peptide TFLLRN (20 μM) to induce cell contraction and phase contrast images were recorded over 50 min. Appearance and increase of substrate folds (white lines, arrowheads) indicate increased cell contraction that was quantified by calculating the image area fraction occupied by wrinkles. (**B**) After 1 h of TFLLRN treatment, cells were immunostained for kindlin-2 (green) and lamin B (red) and confocal images were taken; greyscale insets show kindlin-2. Shown are mean values from at least three independent experiments (data points) ±SD (*** *p* < 0.001, one sample Student *t*-test). Scale bars: 15 µm.

FAs are strained both in response to extracellularly and intracellularly applied force [12,63]. To increase intracellular force, we induced RhoA/Rho-associated kinase-mediated cell contraction using the protease-activated receptor-1 (PAR-1)-activating peptide TFLLRN [64]. When adding TFLLRN to cells grown on “wrinkling” silicone culture substrates, visible surface deformations increased 2.5-fold within 50 min (Figure 4A).

Concomitantly, addition of TFLLRN resulted in a 2.5-fold increase of kindlin-2 protein in the nucleus, compared with vehicle-treated control (Figure 4B). Collectively, three different approaches to mechanically stimulate hCF all demonstrated that kindlin-2 is mechanosensitive and shows enhanced translocation to the nucleus in acutely stressed fibroblasts.

### 3.3. Kindlin-2 Controls α-SMA Promoter Activity during Fibroblast-to-Myofibroblast Activation

**Figure 5:**
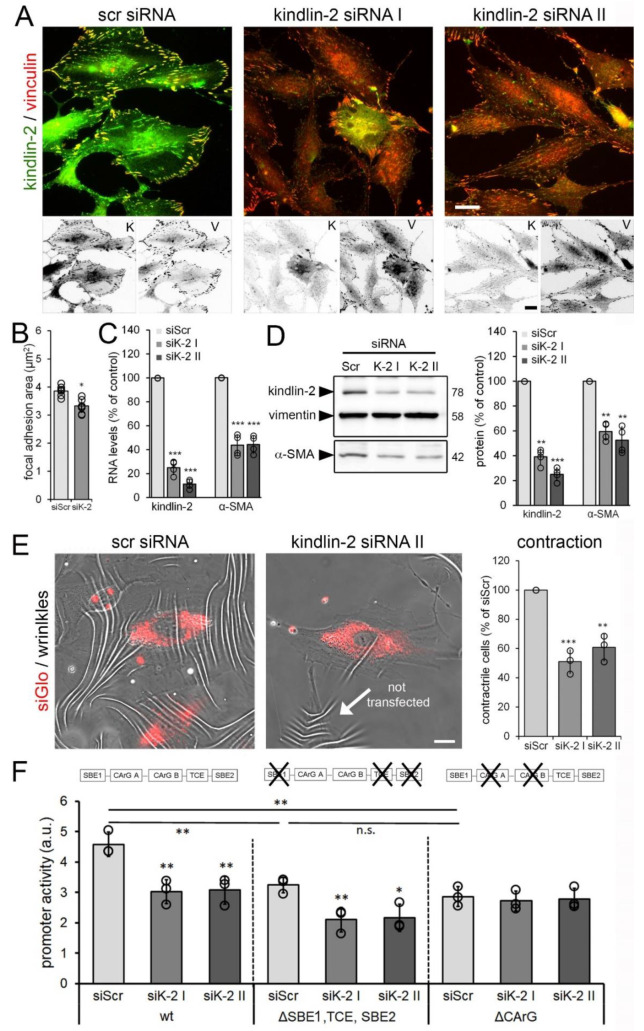
Kindlin-2 knockdown results in reduced myofibroblast activation and contraction. Human cardiac fibroblasts (hCF) were transfected with non-targeting scrambled (scr) and two different silencing RNAs (siRNAs) (siK-2 I & II) targeting the open reading frame of human kindlin-2. After 48 h, cells were analyzed by (**A**) immunostaining for kindlin-2 (K, green and inverted fluorescence) and vinculin (V, red and inverted fluorescence), followed by (**B**) quantification of focal adhesion (FA) sizes based on vinculin signal, (**C**) real-time PCR, and (**D**) quantitative immunoblotting normalized to housekeeping vimentin. (**E**) HCF were co-transfected with siGlo to track transfected cells and kindlin-2-targeting siRNA or non-targeting scrambled siRNA (siScr). Cells were plated 48 h post-transfection onto wrinkling substrates for 8 h to assess and quantify cell contractility (% siGlo-positive cells producing wrinkles). Scale Bars: 15 µm. (**F**) HCF were co-transfected with non-targeting siScr or kindlin-2 siRNA and luciferase reporter plasmids under control of wild-type (wt) α-smooth muscle actin (α-SMA) promoter, α-SMA promoter with inactive Smad-binding elements (SBE) and inactive transforming growth factor (TGF)-β control element (TCE) (ΔSBE1, TCE, SBE2), and α-SMA promoter with inactivated CArGA and CArGB boxes (ΔCArG). Reporter assays were performed 48 h post-transfection and normalized to constitutively expressed renilla luciferase. Shown are mean values from at least three independent experiments (data points) ±SD (* *p* < 0.05, ** *p* < 0.01, *** *p* < 0.005, n.s.-non significant, using ANOVA followed by a post-hoc Tukey’s multiple comparison test).

To test whether kindlin-2 controls myofibroblast activation at the α-SMA promoter level, we downregulated kindlin-2 expression in hCF. HCF transfected with kindlin-2 siRNA remained well spread when assessed after 48 h but exhibited moderately smaller vinculin-positive FAs than control cells (Figure 5A,B).

Kindlin-2 siRNA transfection achieved 60–80% downregulation of kindlin-2, correlating with 40–50% reduction in α-SMA expression both at the protein and mRNA level (Figure 5C,D). Concomitantly, cultures of hCF co-transfected with kindlin-2 siRNAs and siGlo to identify transfected cells by fluorescence microscopy, showed ~2-fold reduced percentages of contractile hCF compared with scrambled siRNA controls (Figure 5E). Hence, kindlin-2 expression contributes to maintaining the α-SMA-positive phenotype and contractile function of cardiac myofibroblasts.

Next, we assessed the possible link between hCF stress perception, nuclear translocation of kindlin-2, and α-SMA gene transcription. Downregulation of kindlin-2 with siRNA resulted in about 30% decrease in luciferase reporter activity under control of the full-length wild-type promoter of α-SMA compared with non-targeting siRNA control (Figure 5F). Kindlin-2 has previously been shown to promote TGF-β1 signaling by binding to the TGF-β1 receptor and the TGF-β1 downstream co-transcription factor Smad3 [57]. To test whether the α-SMA-inducing function of kindlin-2 is mediated via TGF-β1/Smad3, we inactivated the TGF-β control element (TCE) and Smad-binding elements (SBEs) from the α-SMA-promoter in luciferase reporter constructs [45,65,66,67]. Baseline reporter activity of the ∆SBE reporter was 30% lower compared to the wild-type promoter construct (Figure 5F). Knockdown of kindlin-2 reduced the activity of SBE/TCE-inactive α-SMA reporter promoter by 30%, like the wild-type promoter (Figure 5F). Hence, despite the absence of SBE/TCE, loss of kindlin-2 will thus affect α-SMA expression. In addition to SBE/TCE, the α-SMA promoter contains CArG boxes that bind serum response factor (SRF) together with myocardin-related transcription factor (MRTF/MLK-1) [35,67,68,69,70,71]. In fibroblasts, mechanical stimulation and integrin engagement cause nuclear translocation of MRTF-A, which it turn enhances the transcriptional activity of SRF, thereby inducing α-SMA gene expression through the cis-elements [70,72,73,74,75,76,77]. Kindlin-2 knockdown did not reduce luciferase expression under control of α-SMA promoter with inactive CArG boxes below baseline, which was 35% lower compared to the wild-type promoter construct (Figure 5F). These results suggested that kindlin-2 promotes α-SMA transcription via CArG boxes in the α-SMA promoter.

### 3.4. Nuclear Kindlin-2 Plays a Role in Myofibroblast Activation

**Figure 6:**
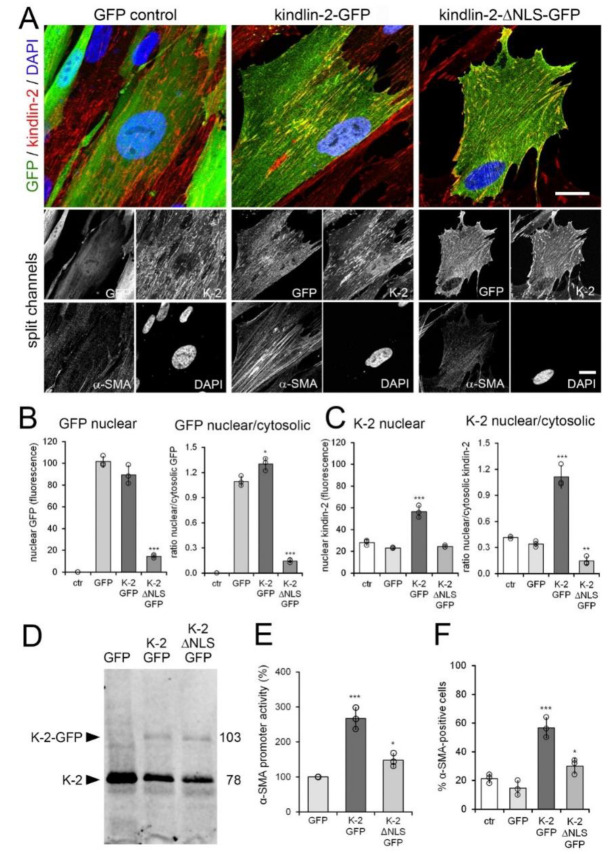
Kindlin-2 overexpression promotes myofibroblast activation. Human MRC-5 fibroblasts were transiently transfected with kindlin-2 (K-2)-GFP, a kindlin-2-GFP mutant lacking the putative nuclear localization sequence (NLS) (kindlin-2-ΔNLS-GFP) and GFP vector control (ctr). Cells were analyzed after 48 h by (**A**,**B**) confocal immunofluorescence co-staining for GFP (green), α-smooth muscle actin (α-SMA) (greyscale), kindlin-2 (red), and 4′,6-diamidino-2-phenylindole (DAPI) (blue) and (**C**,**D**) quantitative immunoblotting against kindlin-2. Scale bar: 25 µm. (**B**) Levels of nuclear and perinuclear (cytosolic) GFP signals were quantified from GFP confocal immunofluorescence images to calculate levels of nuclear GFP and ratios of nuclear/cytosolic GFP only in transfected fibroblasts (green). (**C**) The same was done for kindlin-2 signal, comprising transfected and endogenous kindlin-2. (**D**) Western blot for kindlin-2. (**E**) MRC-5 were additionally transfected with firefly luciferase promoter under full length α-SMA promoter control; signal was first normalized to renilla luciferase and then to GFP control. (**F**) The percentage of α-SMA-positive cells was quantified by manually counting α-SMA stress fiber-positive fibroblasts in the GFP-positive fraction (≥10 cells/image, ≥5 images/experimental condition). Shown are mean values from at least three independent experiments (data points) ±SD (* *p* < 0.05, ** *p* < 0.01, *** *p* < 0.005, using ANOVA followed by a post-hoc Tukey’s multiple comparison test).

Kindlin-2 knockdown resulted in only moderate changes in hCF morphology or FA size after 48 h (Figure 5A,B) despite others reporting severely reduced adhesion and spreading of kindlin-2 deficient cells [78]. Restricting myofibroblast adhesion and FA size has been shown to result in reduced levels of α-SMA expression by reducing intracellular stress [79]. To discriminate between possible adhesion-mediated effects of kindlin-2 in FAs and putative regulation of α-SMA gene transcription by nuclear kindlin-2, we overexpressed kindlin-2-GFP and a kindlin-2-GFP mutant lacking the putative nuclear location sequence (NLS) (kindlin-2-ΔNLS-GFP) in human MRC-5 fibroblasts. MRC-5 were chosen for their low baseline expression of α-SMA and ease of transfection compared to hCF. Both kindlin-2 constructs co-localized with endogenous kindlin-2 in FAs (Figure 6A) and kindin-2-GFP additionally localized to the nucleus and cytosol.

In contrast, kindlin-2-ΔNLS-GFP was almost completely excluded from the nucleus and accumulated in the cytosol (Figure 6A,B), resulting in low ratios of nuclear versus cytosolic kindlin-2 (GFP-tagged plus endogenous) (Figure 6C). Overexpression of kindin-2-GFP resulted in overall enhanced nuclear localization of kindlin-2 over cytosol (Figure 6C).

Transfection rates were comparably low (~25%) (Figure 6D), and strong overexpression in individual cells of both kindlin-2-GFP and kindlin-2-ΔNLS-GFP constructs impacted on the viability of MRC-5 after 3–4 day of culture. Thus, rather than assessing changes in α-SMA protein expression by Western blotting, we co-transfected MRC-5 with kindlin-GFP and kindlin-2-ΔNLS-GFP with luciferase-reporter under control of the full α-SMA promoter (Figure 6E). The promoter activity of α-SMA increased 2.7-fold upon kindlin-2-GFP overexpression compared to 1.5-fold upon kindlin-2-ΔNLS-GFP overexpression (Figure 6E). Promoter activity results after kindlin transfection aligned with the percentage of α-SMA expressing cells among the GFP-positive cells assessed by immunofluorescence (Figure 6F). Transfection with GFP control plasmid did not significantly alter baseline percentage of α-SMA expressing cells (Figure 6F, 14.7 ± 5.0% versus 21.3 ± 3.1%).

**Figure 7:**
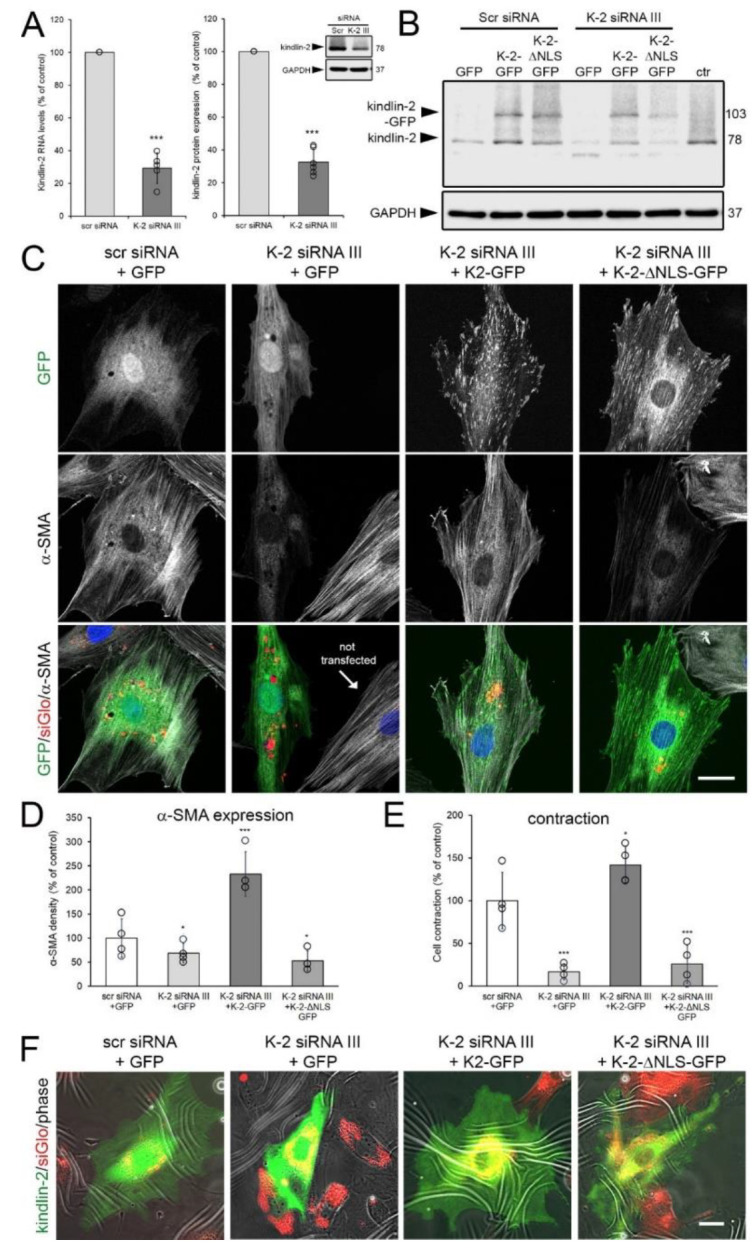
Wild-type but not kindlin-2 missing the putative nuclear localization sequence (NLS) (∆NLS-kindlin-2) rescues myofibroblasts after knockdown of kindlin-2. (**A**) Rat embryonic fibroblasts (REF) were transiently transfected with two 3′-UTR-targeting kindlin2 siRNAs (K-2 siRNA III) and assessed 48 h later using quantitative immunoblotting and qRT-PCR, both normalized to glyceraldehyde 3-phosphate dehydrogenase (GAPDH) loading control. (**B**) Such kindlin knockdown (K-2 siRNA III) or control (scr siRNA) REF were then transfected with plasmid constructs to express GFP, kindlin-2-GFP, and kindlin-2-ΔNLS-GFP and assessed for kindlin-2 expression by immunoblotting, compared to non-transfected REF (ctr). (**C**) Additional transfection with siGlo at the time of knockdown was used to assess expression of GFP constructs (green) and α-smooth muscle actin (α-SMA) (greyscale) in transfected REF (red, siGlo) by confocal microscopy. (**D**) Respective low magnification images were used to quantify the percentage of α-SMA-positive cells by manually counting α-SMA stress fiber-positive fibroblasts in the siGlo/GFP double-positive fraction (≥10 cells/image, ≥5 images/experimental condition). (**E**,**F**) The same approach was used to quantify the percentage of contractile siGlo (red)/GFP (green) double-positive REF grown for 8 h on wrinkling elastomers (wrinkles-white) from epifluorescence images. All graphs show mean values from at least three independent experiments (data points) ±SD (* *p* < 0.05, *** *p* < 0.005, using ANOVA followed by a post-hoc Tukey’s multiple comparison test). Scale bars: 25 µm.

Finally, we assessed the potential of kindlin-2-GFP and kindlin-2-ΔNLS-GFP to rescue myofibroblast features that were lost in rat embryonic fibroblasts (REF) upon kindlin-2 knockdown. Lineage REF were chosen because they exhibit high α-SMA baseline levels like hCF, but are easier to transfect [80]. To be able to rescue knockdown effects and target rat kindlin-2, REF were transfected with a 3′-UTR-targeting kindlin-2 siRNA (K-2 siRNA III). K-2 siRNA III was different from the open reading frame-targeting kindlin-2 siRNA K-2 siRNA I and II used for human fibroblasts. K-2 siRNA III resulted in downregulation of kindlin-2 protein and mRNA, 48 h after transfection of REF (Figure 7A).

Expression of kindlin-2-GFP and kindlin-2-ΔNLS-GFP, transfected into kindlin-2 knockdown cells for another 48 h, was not affected by targeting siRNA or scrambled control siRNA (Figure 7B). Knockdown of kindlin-2 resulted in 1.5-fold reduced density of α-SMA fluorescence signal in REF transfected with scrambled control siRNA compared to REF co-transfected with kindlin-2 siRNA, siGlo transfection indicator, and control GFP (Figure 7C,D). Expression of α-SMA in kindlin-2 knockdown REF was preserved and even enhanced by co-transfection with wild-type kindlin-2-GFP (2.3-fold over control) but not by kindlin-2-ΔNLS-GFP (1.9-fold lower than control) (Figure 7C,D). Confirming earlier results with MRC-5 human fibroblasts, kindlin-2-ΔNLS-GFP was excluded from the cell nucleus but localization to the FAs of REF was not impaired (Figure 7C). Concomitant with α-SMA expression, rescue with wild-type kindlin-2-GFP restored (in fact, enhanced) the contraction of kindlin-2 knocked-down REF on wrinkling elastomer substrates (Figure 7E,F). In contrast, control GFP and kindlin-2-ΔNLS-GFP were not able to rescue reduced contraction in kindlin-2 knockdown cells (Figure 7E,F). In conclusion, localization of kindlin-2 to the cell nucleus is required to contribute to α-SMA expression and myofibroblast activation.

## 4. Discussion

By binding to integrins and the contractile actin cytoskeleton, kindlin-2 is ideally positioned to mediate mechanical signals that are perceived at sites of FAs. From our observation that kindlin-2 expression is upregulated in myofibroblasts upon mechanical overload and fibrosis of the heart, we hypothesized that kindlin-2 regulates the activation of cardiac myofibroblasts. In skin, kindlin-2 is expressed in epidermal keratinocytes and dermal fibroblasts, and it is upregulated upon myofibroblast activation during skin wound healing and in 4–6-week-old human cutaneous scars [42,81]. Kindlin-2 also contributes to kidney fibrosis by interfering with TGF-β1 signaling [58,82]. Our results show that overexpression of kindlin-2 in hCF, MRC-5, and REF results in increased transcription and expression of the myofibroblast marker α-SMA. Consistently, knockdown of kindlin-2 leads to reduced transcription and expression of α-SMA in cultured cardiac myofibroblasts and REF. A major novel finding of our study is that acute mechanical strain induces kindlin-2 translocation into the nuclei of hCF. Applying strain and force locally to FAs using ECM-coated microbeads, globally by stretching hCF on deformable substrates, or by inducing fibroblast contraction all increase kindlin-2 localization in the nucleus within one hour. Although kindlin-2 expression levels were reduced in hCF grown on soft compared to stiff culture substrates, nuclear kindlin-2 levels are comparably low in hCF grown for prolonged periods on stiff culture plastic. Conceivably, kindlin-2 is involved in acute rather than long-term stress responses of fibroblastic cells.

Nuclear accumulation of kindlin-2 has previously been demonstrated in prostate cancer cells [83,84] and smooth muscle cells [85]. Consistently, kindlin-2 contains the putative NLS TKKKKKK within its F1 loop (http://elm.eu.org) [31]. Moreover, kindlin-2 directly interacts with the nuclear shuttle protein, migfilin [86], although nuclear translocation of this complex has not been demonstrated [16,87,88]. Because mechanical strain reduced kindlin-2 association with FAs on microbeads, at least a fraction of kindlin-2 in the nucleus may directly shuttle from FAs. It is tempting to speculate that FA pulling results in conformational changes in β-integrin [89,90,91,92,93,94] to release kindlin-2 from FAs. Mechanosensing mechanisms typically involve protein sensors that are either recruited into FAs or undergo conformational changes in response to mechanical force [12,95,96,97], including vinculin [98], filamin A [41], leukemia-associated Rho guanine nucleotide exchange factor (GEF), and GEF H1 [99], talin1 [100], and p130Cas [62,101,102]. Conformational changes in kindlin-2 have not been described yet.

In contrast to the loss of kindlin-2, talin1 has previously been shown to be recruited to FAs under acute mechanical strain which is possibly explained by their different integrin-binding characteristics [97]. Kindlin-2 directly binds the membrane-distal NxxY motifs of the β1 and β3 integrin cytoplasmic tails via its FERM domain whereas talin1 binds to membrane-proximal NPxY motifs of β-integrin [16,22,26,103,104]. Several studies have indeed revealed distinct roles of talin1 and kindlin-2 in regulating integrin trafficking [34,103,105], integrin force-coupling [106], and cell signaling [97,107]. Given its central role in FAs it is not surprising that kindlin-2 has been implicated in mechanical cell communication with the environment, for instance by integrating the Rho pathway [108,109,110]. Considering our results, it is difficult to appreciate how kindlin-2 can contribute to stress-mediated integrin activation if stress removes it from ECM adhesions. However, our results demonstrate that a substantial fraction of kindlin-2 remains in the stress-bearing peripheral FAs after straining myofibroblasts. Similarly, diverse stress-dependent behavior has been shown for zyxin that is differentially localized in FAs, stress fibers and the nucleus, depending on the levels and location of applied mechanical stress [111,112].

The ability for nuclear translocation seems to be a prerequisite for kindlin-2 to regulate myofibroblast activation. Overexpression of a kindlin-2 mutant lacking the putative NLS at the N-terminus of kindlin-2 resulted in only moderately increased α-SMA transcription in contrast to a strong increase upon wild-type kindlin-2 transfection. Our results suggest that kindlin-2 interacts with factors in the nucleus that are known to control the activity of the α-SMA promoter via CArG boxes, e.g., MRTF and SRF. This novel role of kindlin-2 in transcriptional regulation is consistent with recent findings that kindlin-2 acts as a co-transcription factor with β-catenin and T-cell factor 4 to control transcription genes regulated by the Wnt pathway, including axin-2, cyclin D1, twist, lymphoid enhancer-binding factor 1, matrix metalloproteinase-2, secreted frizzled-related protein, and versican [84]. Moreover, kindlin-2 was recently shown to control renal tubular epithelial-to-mesenchymal differentiation via ERK1/2 and Akt signaling pathways [58]. Epithelial-to-mesenchymal transition is the first step in a process that ultimately culminates in the expression of α-SMA as a key indicator of the “myogenic” differentiation program under the conditions of tissue repair and fibrosis [113]. Another important transcriptional co-factor that was shown to regulate myofibroblast activation is MRTF-A in conjunction with SRF [70,73,74,114]. Conceivably, kindlin-2 primarily senses the state of force transduction through integrins whereas MRTF-A senses the state of actin polymerization; these two types of information may then converge on SRF to regulate transcription of the α-SMA promoter.

It is unlikely that kindlin-2 downregulation and overexpression affect myofibroblast activation exclusively due to a nuclear function. The same poly lysine motif in kindlin-2 that putatively controls nuclear localization was reported to promote binding of the F1 loop in kindlin to acidic membrane phospholipids and cooperation with talin1 in integrin activation [115]. However, our deletion mutant was able to recruit to FAs, in contrast to the mutant used in this previous study [115], and we did not observe dramatic loss of adhesion or alteration of vinculin-positive FAs in 48 h kindlin-2 knockdown experiments with three different fibroblasts: hCF, REF, and MRC-5. It is possible that adhesion of cells with low kindlin-2 levels is rescued by integrins that are less dependent on kindlin-2-mediated regulation. For instance, αvβ5 integrin is expressed in cardiac fibroblasts [51] and promotes cell spreading and adhesion to fibronectin and vitronectin among other ECM ligands. Recent studies suggest that kindlin-2 may not be required to bind to αvβ5 integrin to promote cell adhesion [116]. Nevertheless, it is likely that kindlin-2 expression levels will influence activation of other integrins (e.g., α5β1 integrin) and fibroblast adhesion strength [117] as shown by reduced spreading of suspended kindlin-2 knockdown cells [78]. The reduced ability of hCF and REF to wrinkle deformable substrates is possibly a combination of reduced force transmission due to changes in FAs and/or reduced contraction due to the loss of α-SMA in stress fibers. Our own research has shown that both are crucial in promoting long term myofibroblast activation [51,79,80].

We conclude that both functions of kindlin-2, as a novel mechanosensor that shuttles to the nucleus of mechanically strained cells and for FA protein-promoting integrin activation, have a central role in controlling myofibroblast activation. Whether this role makes kindlin-2 a suitable, i.e., sufficiently specific, target to treat conditions of fibrosis remains to be shown.

## Supplementary Materials

The following are available online at https://www.mdpi.com/2073-4409/9/12/2702/s1, Video S1: Human cardiac fibroblasts were incubated with fibronectin-coated magnetic microbeads for two hours. Cells were then fixed permeabilized, and immunostained for kindlin-2 (green) and lamin B (red). Z-stacks of confocal optical sections were obtained by structured illumination (Apotome, Zeiss) with 0.1 µm oversampling (left upper frame moves through the 114 sections) and used to reconstruct three-dimensional images. Reconstructions of kindlin-2 alone (lower left), lamin B alone (lower right) and merged channels (upper right) are rotated by 180°. Video S2: Human cardiac fibroblasts were incubated with fibronectin-coated magnetic microbeads for one hour followed by tensile force application using a magnet for another hour. Cells were then fixed permeabilized, and immunostained for kindlin-2 (green) and lamin B (red). Z-stacks of confocal optical sections were obtained by structured illumination (Apotome, Zeiss) with 0.1 µm oversampling (left upper frame moves through the 120 sections) and used to reconstruct three-dimensional images. Reconstructions of kindlin-2 alone (lower left), lamin B alone (lower right) and merged channels (upper right) are rotated by 180°.
